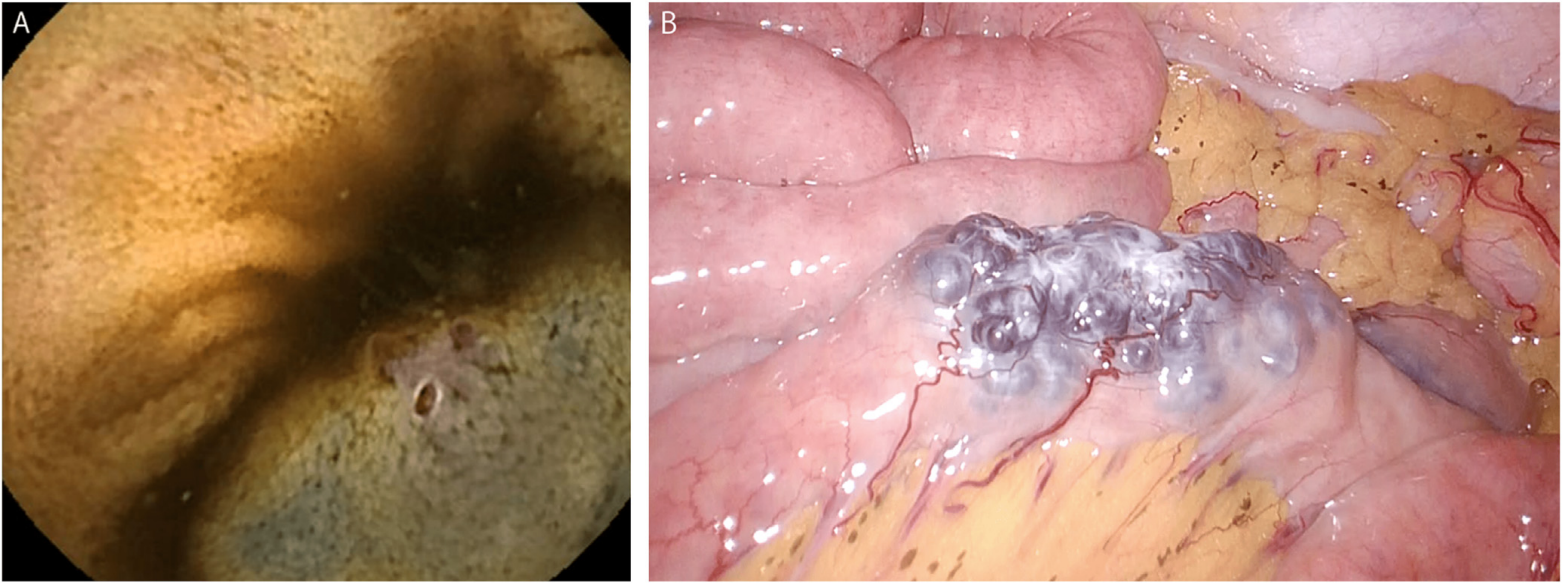# Repeated Critical Hemorrhage Cured by Small Bowel Enteroscopy and Laparoscopic Surgery

**DOI:** 10.1016/j.gastha.2024.05.005

**Published:** 2024-05-24

**Authors:** Yasutaka Saito, Sumito Sato

**Affiliations:** Department of Surgery, Seirei Hamamatsu General Hospital, Shizuoka, Japan

A 57-year-old male patient presented to the emergency department with hematochezia. His medical history was significant for multiple episodes of hematochezia requiring blood transfusions, the cause of which remained undetermined despite endoscopic examinations. He was also receiving antiplatelet therapy.

Despite undergoing contrast-enhanced computed tomography and upper and lower gastrointestinal endoscopy, no source of bleeding was identified. However, capsule endoscopy revealed a mucosal discoloration in the small intestine, suggesting a potential bleeding site (Figure A). As no lesions were detected during flexible fiberoptic proximal/distal small bowel enteroscopy, a marking tattoo injection was performed to outline the area of interest, followed by surgical intervention.

During the surgery guided by the tattoo, a 6 cm lesion in the jejunum with noticeable vascular dilation was identified laparoscopically (Figure B), and a partial resection was performed. A pathological examination revealed numerous dilated veins extending from the submucosal to the subserosal layers, along with a few small arteries, confirming the diagnosis of small bowel arteriovenous malformation.

Arteriovenous malformation of the small bowel is a rare condition and can pose diagnostic challenges. A combined approach of small bowel enteroscopy and laparoscopy is beneficial for suspected small bowel bleeding when the lesion is unidentified and enables minimally invasive surgery.

**Figure:**